# The Effects of Female Sexual Hormones on the Expression of Aquaporin 5 in the Late-Pregnant Rat Uterus

**DOI:** 10.3390/ijms17081300

**Published:** 2016-08-22

**Authors:** Adrienn Csányi, Judit Bóta, George Falkay, Robert Gáspár, Eszter Ducza

**Affiliations:** Department of Pharmacodynamics and Biopharmacy, Faculty of Pharmacy, University of Szeged, H-6720 Szeged, Hungary; csanyi.adrienn@pharm.u-szeged.hu (A.C.); bota.judit@pharm.u-szeged.hu (J.B.); falkay@pharm.u-szeged.hu (G.F.); gaspar@pharm.u-szeged.hu (R.G.)

**Keywords:** aquaporin 5, pregnancy, estrogen, gestagen

## Abstract

Thirteen mammalian aquaporin (AQP) water channels are known, and few of them play a role in the mammalian reproductive system. In our earlier study, the predominance of AQP5 in the late-pregnant rat uterus was proven. Our current aim was to investigate the effect of estrogen- and gestagen-related compounds on the expression of the AQP5 channel in the late-pregnant rat uterus. Furthermore, we examined the effect of hormonally-induced preterm delivery on the expression of AQP5 in the uterus. We treated pregnant Sprague-Dawley rats subcutaneously with 17β-estradiol, clomiphene citrate, tamoxifen citrate, progesterone, levonorgestrel, and medroxyprogesterone acetate. Preterm delivery was induced by subcutaneous mifepristone and intravaginal prostaglandin E2. Reverse-transcriptase PCR and Western blot techniques were used for the detection of the changes in AQP5 mRNA and protein expressions. The amount of AQP5 significantly increased after progesterone and progesterone analogs treatment on 18 and 22 days of pregnancy. The 17β-estradiol and estrogen receptor agonists did not influence the AQP5 mRNA level; however, estradiol induced a significant increase in the AQP5 protein level on the investigated days of gestation. Tamoxifen increased the AQP5 protein expression on day 18, while clomiphene citrate was ineffective. The hormonally-induced preterm birth significantly decreased the AQP5 level similarly to the day of delivery. We proved that AQP5 expression is influenced by both estrogen and progesterone in the late-pregnant rat uterus. The influence of progesterone on AQP5 expression is more predominant as compared with estrogen.

## 1. Introduction

Aquaporins (AQPs) are small hydrophobic integral membrane proteins which facilitate rapid passive movement of water across the cell membrane [1]. To this day, thirteen AQP isoforms have been identified in mammals, and twelve of them can be found in female and male reproductive tissues in mice, rats, dogs, swine, and humans [2,3]. According to the coding sequence and the permeability features, AQPs are divided into three major subtypes: classical AQPs, aquaglyceroporins, and unorthodox aquaporins. AQP0, 1, 2, 4, 5, and 8 are classical AQPs which are water-selective channels, while AQP3, 7, 9, and 10 can be classified as aquaglyceroporins and are permeable to glycerol, urea, and other small solutes, as well as water [4,5]. AQP11 and 12 belong to the unorthodox aquaporin group, the function of which has not been clearly identified [6,7].

AQPs have an essential role in the female reproductive system, and in the mammalian uterus the AQP1, 2, 3, 4, 5, 7, 8, and 9 isoforms have been detected [2]. The AQP1, 2, 3, and 8 isoforms might be involved in water movement during uterine imbibition, and they may play important roles in hormone-mediated water and other small molecule transport [8]. AQP8 knockout (KO) pregnant mice had a significantly higher number of embryos, and fetal/neonatal weight and the amount of amniotic fluid was greater compared to wild type controls [9]. Contrarily, the number of embryos and the fetal weight decreased in AQP1-KOs. The AQP1-KO placenta demonstrated increased degeneration with evidence of altered blood vessel structure and increased syncytiotrophoblast nodules [10]. AQP5-KO female mice did not show any difference in conception and implantation as compared with wild-type animals [11]. He et al. [12] found that AQP2 expression in human endometrium correlates with serum 17β-estradiol and progesterone levels; therefore, it is menstrual cycle-dependent. Former studies identified the estrogen response element in the promoter region of the AQP2 and 5 genes, which provides the immediate regulation of these water channels by estrogen [13,14]. Water channel regulation was also investigated in the uterus of cycling bitches, and it was discovered that AQP5 was particularly expressed in response to high levels of progesterone [15]. Skowronska et al. [16] also investigated the changes of AQP5 expression due to progesterone and estradiol treatments in an in vitro study on porcine uterus. They found that AQP5 gene expression was down-regulated after the estrogen and progesterone treatments during the mid-luteal phase, but during luteolysis, it was increased by estrogen. A recent experiment was carried out on ovariectomized rats, and they received testosterone, estrogen, or a combination of them. This study determined that testosterone enhanced the expression of AQP5 in the uterus, and this effect was diminished by a following estrogen treatment [17]. They also observed the increase of AQP5 protein expression in ovariectomized rat uterus in response to progesterone alone or in combination with estrogen [18].

It has been postulated that AQPs take part in the processes of fertilization, blastocyst formation, and implantation [19]. AQP1 and 2 are the most concentrated in the endometrium at the time of implantation, suggesting that they may have a physiological role in uterine receptivity [12,20]. Moreover, the cellular and subcellular localizations of amniotic AQPs indicate that the AQPs play distinct functional roles, such as apoptosis for amniotic fluid homeostasis or the tissue remodeling of amniotic membranes during pregnancy. It was proven that AQP1, 3, 8, 9, and 11 play crucial roles in the transfer of water across the placenta [21].

In earlier studies, we determined the expression of AQP1, 2, 3, 5, 8, and 9 isoforms in the pregnant rat uterus. We observed that AQP5 mRNA and protein expression showed the most significant changes during pregnancy. AQP5 expression was the most remarkable on days 18–21 of pregnancy and dramatically dropped on the last day (day 22) of gestation. We determined the effect of oxytocin on the myometrial expression of AQP5. Our results led us to suppose that oxytocin selectively decreases the expression of AQP5 at the end of pregnancy and may be of importance in the initiation of delivery in rats [22].

It is well known that the expression of various AQPs can be regulated by steroid sex hormones [18,23] in female reproductive tissues, but we have limited data concerning the expression of AQP5 under the effect of female sexual hormones in the late-pregnant uterus and the delivery. The primary aim of this study was to investigate the effects of estrogen and progesterone receptors agonists on the expression of AQP5 in the late-pregnant rat uterus. We also wanted to investigate the alteration of uterine AQP5 expression during hormonally-induced preterm birth in rats.

## 2. Results

### 2.1. The Effects of Estrogen-Related Compounds on Aquaporin 5 (AQP5) Expression

**Figure 1:**
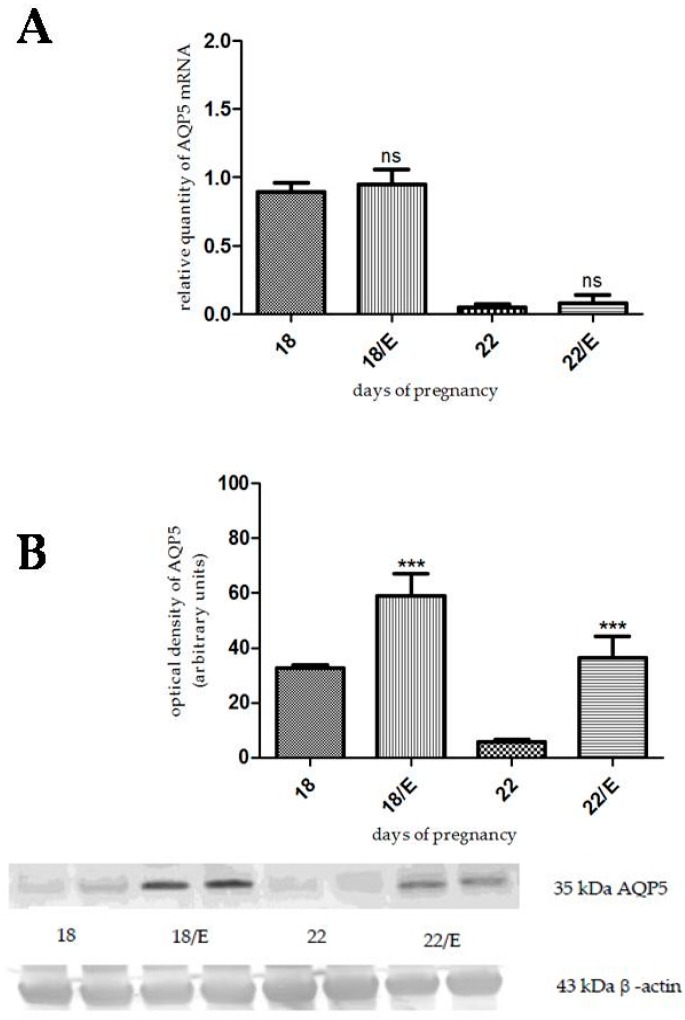
Results of PCR and Western immunoblotting experiments after 17β-estradiol treatment. The changes of mRNA (**A**) and protein (**B**) expression of aquaporin 5 (AQP5) after 17β-estradiol (E) pretreatment in pregnant rat uterus on days 18 and 22. ns > 0.05, *** *p* < 0.001 as compared with the data on non-treated control uterus. Each bar denotes the mean ± S.D. *n* = 6. The bands resulting from non-specific bindings of the polyclonal AQP5-antibody were not changed by 17β-estradiol treatment.

We investigated the effect of 17β-estradiol on AQP5 expression on day 18 and 22 of pregnancy. 17β-estradiol pretreatment did not cause any significant changes in the AQP5 mRNA levels, either on day 18 or day 22 of pregnancy compared to the control (Figure 1A). The protein expression of AQP5 significantly increased both on day 18 (*p* < 0.001) and day 22 (*p* < 0.001) of pregnancy, compared to the non-treated control uterus (Figure 1B).

**Figure 2:**
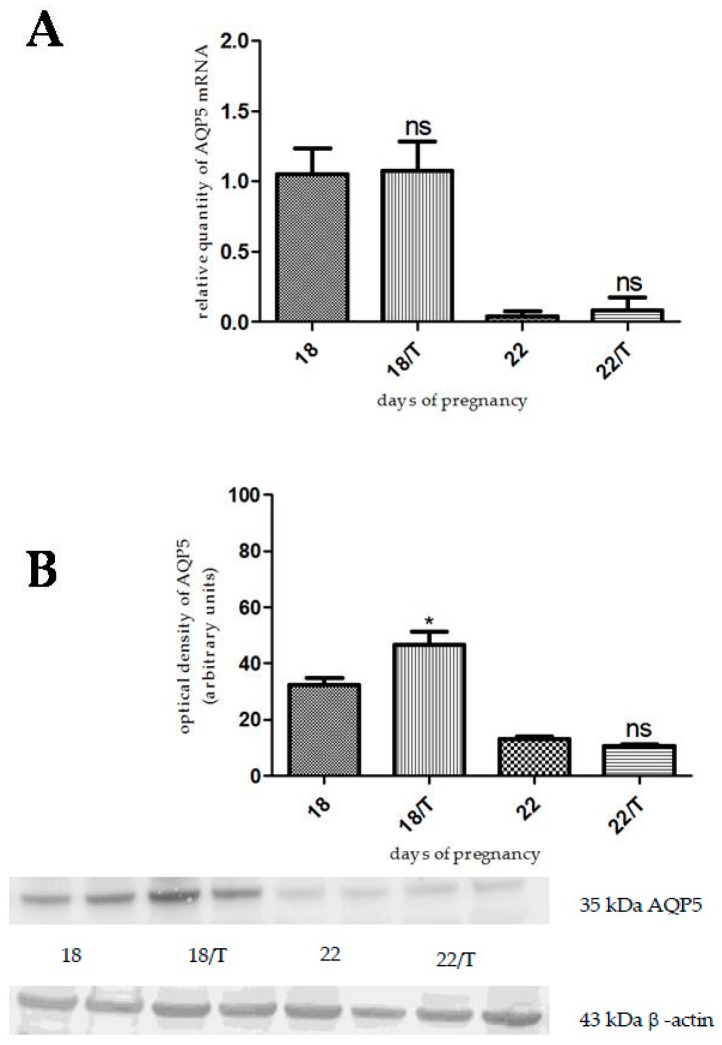
Results of PCR and Western immunoblotting experiments after tamoxifen treatment. The changes of mRNA (**A**) and protein (**B**) expression of AQP5 after tamoxifen citrate (T) pretreatment in pregnant rat uterus on days 18 and 22. ns > 0.05, * *p* < 0.05 as compared with the data on non-treated control uterus. Each bar denotes the mean ± S.D. *n* = 6. The bands resulting from non-specific bindings of the polyclonal AQP5-antibody were not changed by tamoxifen treatment.

The AQP5 mRNA expression did not change as a result of tamoxifen citrate pretreatment on the investigated days (Figure 2A). In contrast, the level of AQP5 protein increased on day 18 (*p* < 0.0124), but did not change on the last day of pregnancy, compared to the control (Figure 2B).

**Figure 3:**
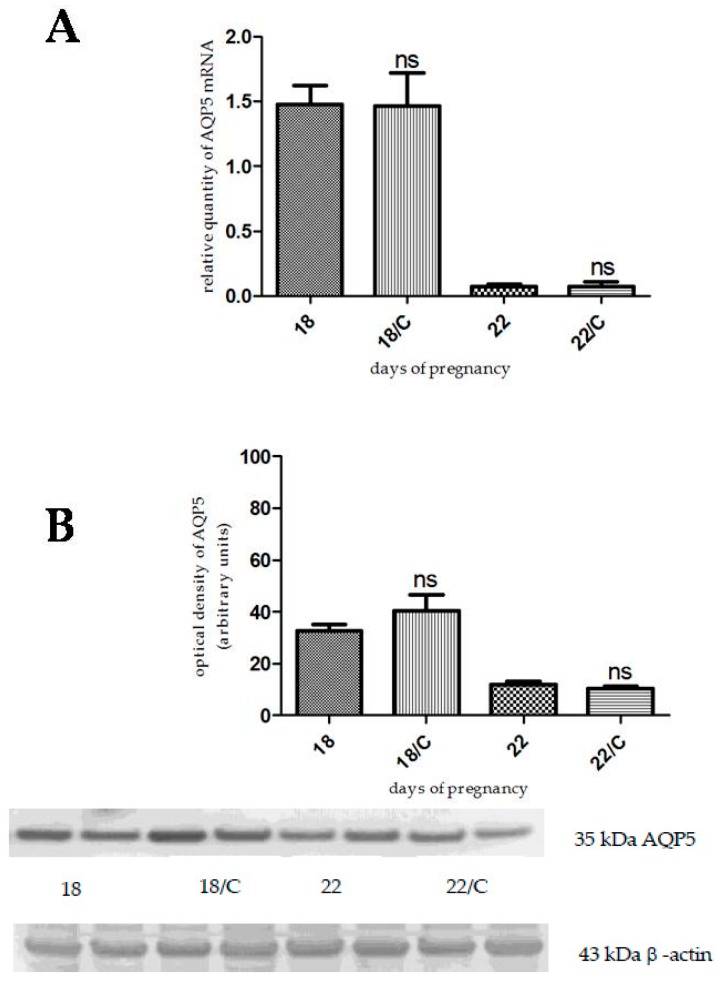
Results of PCR and Western immunoblotting experiments after clomiphene citrate treatment. The changes of mRNA (**A**) and protein (**B**) expression of AQP5 after clomiphene citrate (C) pretreatment in pregnant rat uterus on days 18 and 22. ns > 0.05 as compared with the data on non-treated control uterus. Each bar denotes the mean ± S.D. *n* = 6. The bands resulting from non-specific bindings of the polyclonal AQP5-antibody were not changed by clomiphene citrate treatment.

Clomiphene citrate pretreatment did not cause any significant changes in the AQP5 mRNA (Figure 3A) and protein (Figure 3B) levels on day 18 and day 22 of pregnancy, compared to the control.

### 2.2. The Effects of Gestagen-Related Compounds on AQP5 Expression

**Figure 4:**
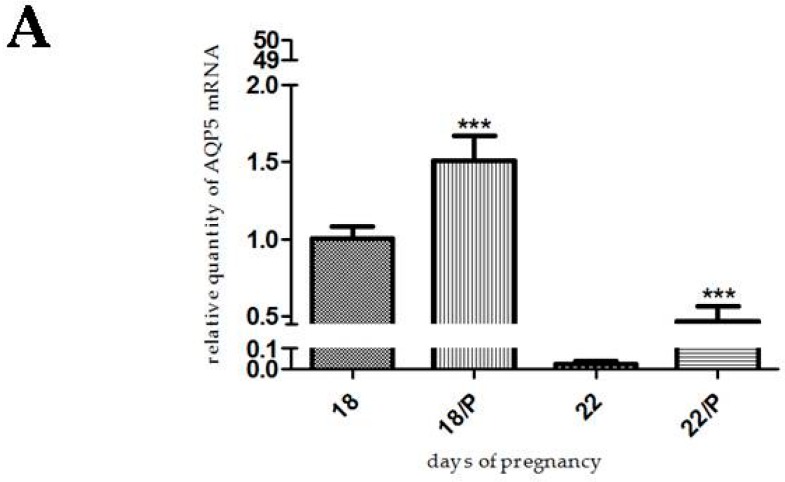

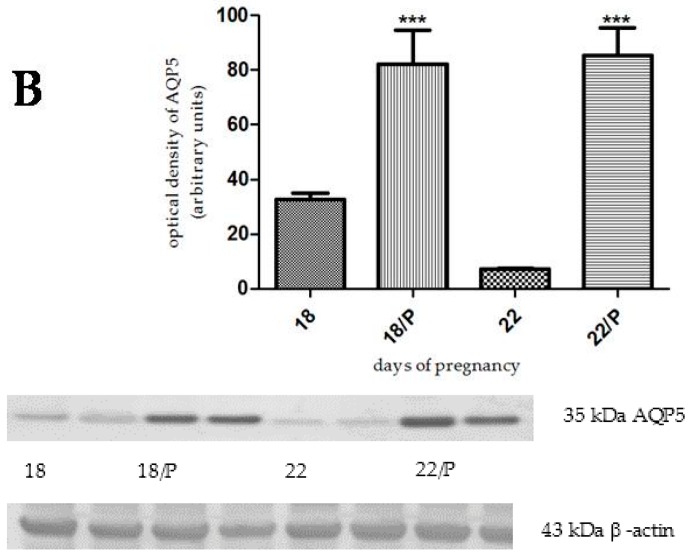
Results of PCR and Western immunoblotting experiments after progesterone treatment. The changes of mRNA (**A**) and protein (**B**) expression of AQP5 after progesterone (P) pretreatment in pregnant rat uterus on days 18 and 22. *** *p* < 0.001 as compared with the data on non-treated control uterus. Each bar denotes the mean ± S.D. *n* = 6. The bands resulting from non-specific bindings of the polyclonal AQP5-antibody were not changed by progesterone treatments.

**Figure 5:**
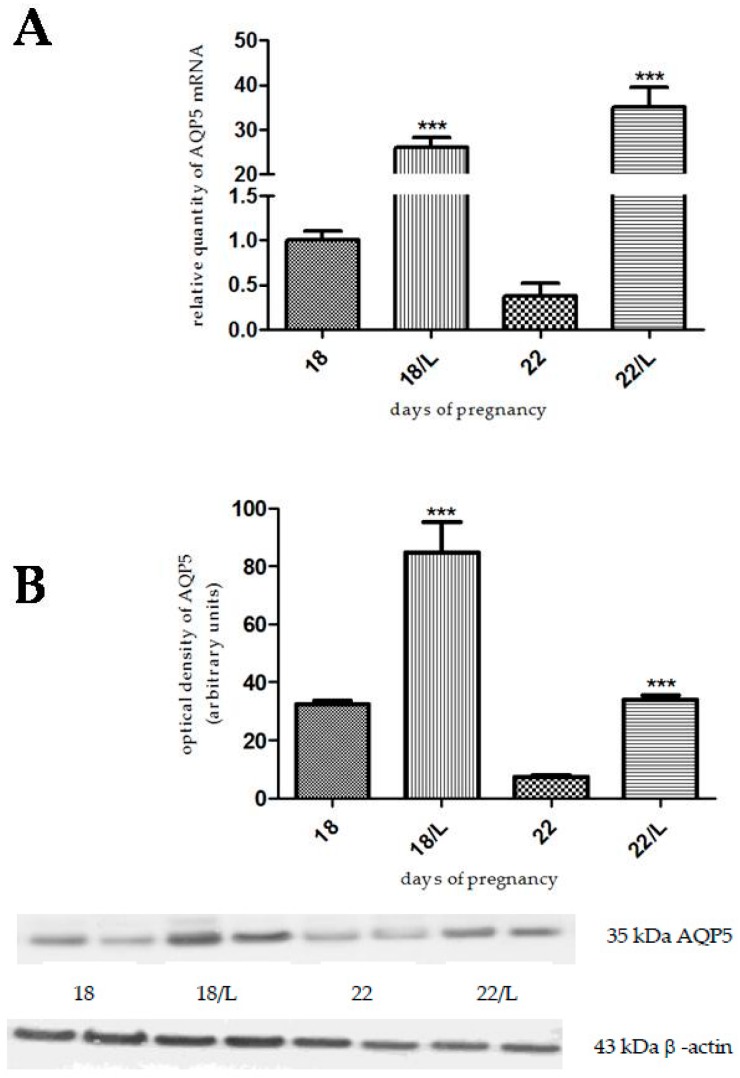
Results of PCR and Western immunoblotting experiments after levonorgestrel treatment. The changes of mRNA (**A**) and protein (**B**) expression of AQP5 after levonorgestrel (L) pretreatment in pregnant rat uterus on days 18 and 22. *** *p* < 0.001 as compared with the data on non-treated control uterus. Each bar denotes the mean ± S.D. *n* = 6. The bands resulting from non-specific bindings of the polyclonal AQP5-antibody were not changed by levonorgestrel treatments.

**Figure 6:**
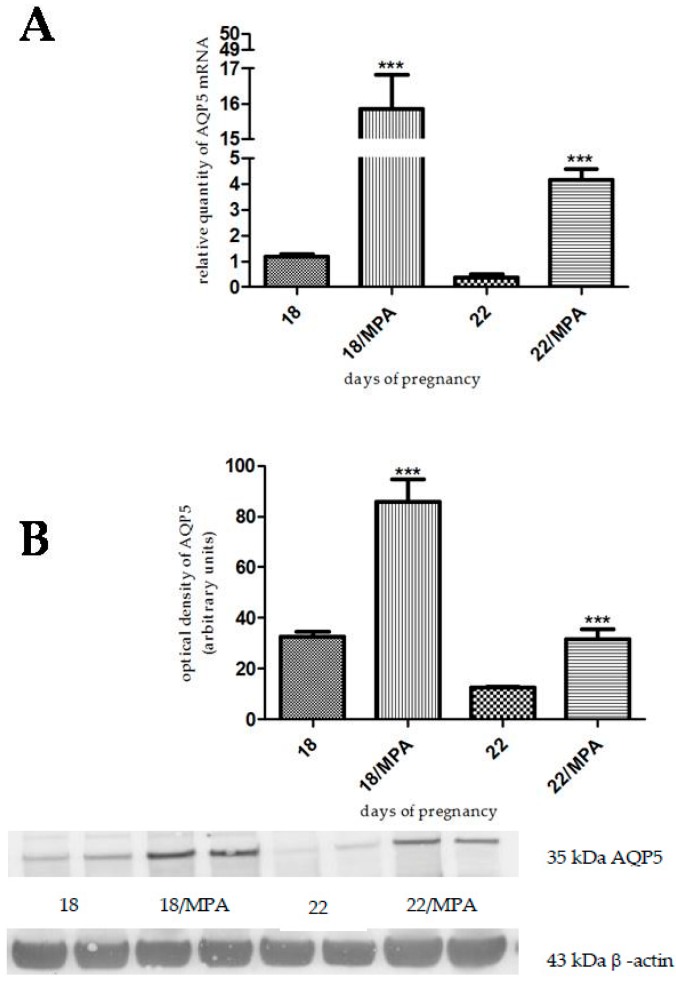
Results of PCR and Western immunoblotting experiments after medroxyprogesterone acetate treatment. The changes of mRNA (**A**) and protein (**B**) expression of AQP5 after medroxyprogesterone acetate (MPA) pretreatment in pregnant rat uterus on days 18 and 22. *** *p* < 0.001 as compared with the data on non-treated control uterus. Each bar denotes the mean ± S.D. *n* = 6. The bands resulting from non-specific bindings of the polyclonal AQP5-antibody were not changed by MPA treatments.

Progesterone, levonorgestrel, and medroxyprogesterone acetate pretreatment caused a significant increase in AQP5 mRNA and protein levels both on day 18 (*p* < 0.001) and day 22 (*p* < 0.001) of pregnancy (Figure 4, Figure 5 and Figure 6).

### 2.3. The Effect of Hormonally-Induced Preterm Delivery on AQP5 Expression

**Figure 7:**
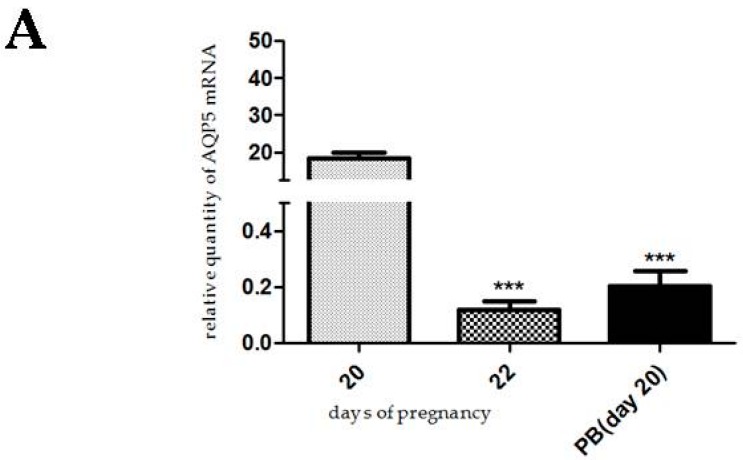

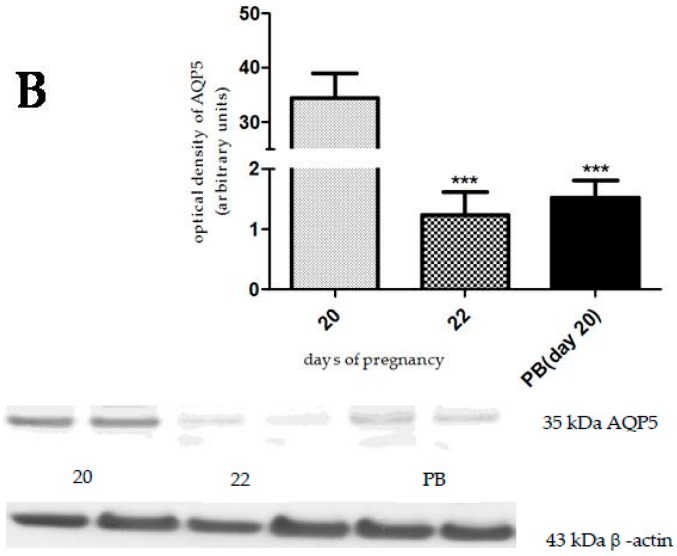
Results of PCR and Western immunoblotting experiments after hormonally-induced preterm delivery. The changes of mRNA (**A**) and protein (**B**) expression of AQP5 after the hormonally-induced preterm birth (PB) in pregnant rat uterus and non-treated pregnant rat uterus. *** *p* < 0.001 as compared with the data on non-treated pregnancy day 20. Each bar denotes the mean ± S.D. *n* = 6. The bands resulting from non-specific bindings of the polyclonal AQP5-antibody were not changed by hormonal treatments.

In the hormonally-induced preterm delivery model, a significant decrease of AQP5 mRNA and protein levels was caused on day 20 of pregnancy, compared to the non-treated animals on pregnancy day 20 (*p* < 0.001). This decrease in AQP5 expression was similar to the last day of pregnancy (Figure 7).

## 3. Discussion

AQPs are detected in the female reproductive tissues, and it has been revealed that they are involved in embryo implantation and endometrial development during pregnancy [2]. In our earlier studies, it was determined that AQP5 expression shows the highest value on day 18 of pregnancy, and a remarkable decrease was found on the last day of pregnancy in rats [22].

It is a known fact that characteristic hormonal changes take place during pregnancy. In humans, the levels of estrogen and progesterone show a constantly growing trend during gestation. At the end of pregnancy the amount of estrogen in blood remains high, while the serum progesterone level moderately declines [24,25]. In pregnant rats, the level of estradiol is constant, and then doubles from day 18 until day 21 of gestation. On the last day of pregnancy, the amount of estradiol shows a slight decrease [26]. The progesterone level increases continuously during pregnancy in rats until day 19 of gestation, at which point it decreases dramatically [27].

It is well known that the expressions of water channels are influenced by sexual hormones. Jablonski et al. [8] proved that the AQP1 channel was slightly estrogen-regulated in the non-pregnant mouse uterus, and that the expression of AQP2 was significantly stimulated by estrogen. Another study confirmed that AQP1, 4, and 5 are excessively expressed in the periimplantation period in mouse uterus, and that AQP5 expression depends on the estrogen stimulation of the progesterone-primed uterus [19]. In the early days of pregnancy, the location of the AQP5 channel changes from the cytoplasm to the apical plasma membrane [20].

As seen in the foregoing, there is limited information about the hormonal effects on AQP5 expression in the pregnant rat uterus. In the present study, we determined a significant increase in AQP5 expression after progesterone pretreatment in the late-pregnant rat uterus. We investigated two progesterone analogs, because levonorgestrel and medroxyprogesterone acetate have a relatively good binding affinity on the progesterone receptor [28,29]. The increase in AQP5 expression was higher in the mRNA level, but the protein expression showed a similar increase after levonorgestrel and medroxyprogesterone acetate treatment, as compared with progesterone treatment. The central concept of molecular biology deals with the transfer of information from DNA via mRNA to proteins. Several biological factors were identified which influence this process, so we cannot always prove a strong correlation between mRNA and protein expression [30]. We suppose that estrogen may increase the stability of AQP5 protein without alteration of mRNA expression, and that this process can lead to the increase in protein expression.

In our study, 17β-estradiol pretreatment did not cause any changes in the AQP5 mRNA expression, but the amount of AQP5 protein was significantly increased, both on day 18 and day 22 of pregnancy. In view of this, it appears that estrogen has a significant effect on AQP5 protein expression in the late-pregnant rat uterus.

Tamoxifen citrate is a nonsteroidal triphenylethylene derivative with a selective estrogen receptor modulator effect. This means that tamoxifen acts like antiestrogen in breast but estrogen agonist in bone and uterus, and it is used in the treatment of certain estrogen-dependent breast cancer [31]. In the case of tamoxifen citrate, there was a significant increase in the amount of AQP5 protein on day 18 of pregnancy.

Clomiphene citrate is a nonsteroidal drug which also has selective estrogen receptor modulator effects. It consists of two isomers which have mixed estrogenic and antiestrogenic effects, and these features depend on the target tissue [32]. Clomiphene citrate did not cause any changes either in the AQP5 mRNA expression or in the AQP5 protein amount. We suppose that clomiphene could not exert its effect on the hypothalamic-pituitary axis during the four-day-long pretreatment.

There are different types of animal models for preterm birth, since preterm delivery could have various pathophysiological backgrounds. We used a hormonally-induced model to prove the progesterone effect on AQP5 expression. Preterm delivery was induced by antigestagen mifepristone and prostaglandin E2. Mifepristone induces preterm delivery by blocking the progesterone receptor [33]. Prostaglandin E2 contributes to cervical ripening and the induction of labor [34]. In this model, day 20 was the starting day of preterm birth. We found a marked decrease of AQP5 mRNA and protein levels on day 20, and this change in AQP5 expression was similar to the last day (day 22) of gestation. The explanation for this phenomenon could be that in the preterm delivery, the progesterone level decreases significantly, followed by the reduction of AQP5 expression.

In summary, we can conclude that AQP5 expression is influenced by both female hormones with progesterone predominance in the late-pregnant rat uterus. Our preterm birth model studies suggest that the lack of progesterone effect leads to reduced AQP5 expression and may contribute to the initiation of preterm labor.

## 4. Materials and Methods

### 4.1. Experimental Animals

#### 4.1.1. Housing and Handling of the Animals

The animals were treated in accordance with the European Communities Council Directive (86/609/ECC) and the Hungarian Act for the Protection of Animals in Research (Article 32 of Act XXVIII), and all experiments involving animal subjects were carried out with the approval of the Hungarian Ethical Committee for Animal Research (permission number: IV/198/2013).

Sprague-Dawley rats (INNOVO Ltd., Gödöllő, Hungary) were kept at a controlled temperature of 20–23 °C, in relative humidity of 40%–60% and under a 12 h light/dark cycle. The animals were fed a standard rodent pellet diet (INNOVO Ltd., Isaszeg, Hungary), with tap water available ad libitum.

#### 4.1.2. Mating of the Animals

Mature female Sprague-Dawley rats (180–200 g) in estrus were collected. The vaginal impedance was measured by an Estrus Cycle Monitor EC40 (Fine Science Tools, Foster City, CA, USA). The appropriate female and male rats (240–260 g) were mated in a special mating cage. In this cage there was a time-controlled movable metal door separating the rats of different sex. Since rats are usually active at night, the separating door was opened in the early morning hours. Four or five hours after the potential copulation, vaginal smears were taken from the female rats and were examined by a microscope at a 1200× magnification. The presence of a copulation plug or the presence of sperm in the native vaginal smear was accepted as proof of the mating. These female animals were separated and regarded as first-day pregnant rats [22,35].

### 4.2. In Vivo Treatments of the Rats

The 17β-estradiol valerate, tamoxifen citrate, and clomiphene citrate (Sigma-Aldrich, Budapest, Hungary) pretreatment of the pregnant animals was started on day 14 and day 18 of pregnancy. The compounds were dissolved in olive oil. The animals were injected subcutaneously with 1 µg/0.1 mL of 17β-estradiol [35,36], 5 mg/0.1 mL of tamoxifen citrate [37], and 1 mg/0.1 mL of clomiphene citrate [38,39] once a day for four days. On day 18 and 22, the uterine samples were collected and the molecular studies were carried out.

The progesterone, levonorgestrel, and medroxyprogesterone acetate (Sigma-Aldrich) pretreatment of the pregnant animals was started on day 11 and 15 of pregnancy. These hormones were dissolved in olive oil and injected subcutaneously every day in a dose of 0.5 mg/0.1 mL of progesterone [35,36] and levonorgestrel [40], and 5 mg/0.1 mL of medroxyprogesterone acetate [41] for seven days. On day 18 and 22, the uterine samples were collected, and the molecular studies were carried out. The duration of the hormonal treatments were determined based on our earlier studies [35,42,43].

The preterm delivery group was treated with mifepristone (Sigma-Aldrich), which was dissolved in olive oil and given subcutaneously in a dosage of 3 mg/0.1 mL on day 19 of pregnancy at 9 A.M. On the same day at 4 P.M., the animals received intravaginal prostaglandin E2 at a dose of 0.5 mg/mL [44]. The preterm birth occurred on the following day (day 20 of pregnancy). Uterine samples were collected after the beginning of the preterm birth, and molecular studies were carried out.

### 4.3. RT-PCR Studies

#### 4.3.1. Tissue Isolation

The rats (250–300 g) were sacrificed by CO_2_ inhalation. Newborn rats were sacrificed by immediate cervical dislocation. The uterine tissues from pregnant animals (*n* = 6) (tissue between two implantation sites) were rapidly removed and placed into RNAlater Solution (Sigma-Aldrich). The tissues were frozen in liquid nitrogen and stored at −75 °C until the extraction of total RNA.

#### 4.3.2. Total RNA Preparation

Total cellular RNA was isolated by extraction with guanidinium thiocyanate-acid-phenol-chloroform according to the procedure of Chomczynski and Sacchi [45]. After precipitation with isopropanol, the RNA was washed with 75% ethanol and then re-suspended in diethyl pyrocarbonate-treated water. RNA purity was controlled at an optical density of 260/280 nm with BioSpec Nano (Shimadzu, Kyoto, Japan); all samples exhibited an absorbance ratio in the range of 1.6–2.0. RNA quality and integrity were assessed by agarose gel electrophoresis.

#### 4.3.3. Real-Time Quantitative Reverse-Transcriptase PCR

Reverse transcription and amplification of the PCR products were performed by using the TaqMan RNA-to-*C*_T_-Step One Kit (Life Technologies, Budapest, Hungary) and an ABI StepOne Real-Time cycler. Reverse-transcriptase PCR amplifications were performed as follows: 48 °C for 15 min and 95 °C for 10 min, followed by 40 cycles at 95 °C for 15 s and 60 °C for 1 min. The samples of qPCR experiments contained “no-template” control, “absolute” control or RNA samples from non-treated and treated uterus. The generation of specific PCR products was confirmed by melting curve analysis. Table 1 contains the assay IDs for the primers used and the reaction parameters. All samples were run in triplicate. The fluorescence intensities of the probes were plotted against PCR cycle number. The amplification cycle displaying the first significant increase of the fluorescence signal was defined as the threshold cycle (*C*_t_).

### 4.4. Western Blot Analysis

The uterine tissues from pregnant animals (tissue between two implantation sites) were homogenized using a Micro-Dismembrator (Sartorius AG, Goettingen, Germany) and centrifuged at 5000× *g* for 15 min at 4 °C in RIPA Lysis Buffer System (Santa Cruz Biotechnology, Inc., Dallas, TX, USA), which contains phenylmethylsulfonyl fluoride (PMSF), sodium orthovanadate, and protease inhibitor cocktail. Total protein amounts from supernatant were determined with spectrophotometry (BioSpec-nano, Shimadzu, Japan).

Twenty-five micrograms of sample protein per well was subjected to electrophoresis on 4%–12% NuPAGE Bis-Tris Gel in XCell SureLock Mini-Cell Units (Life Technologies). Proteins were transferred from gels to nitrocellulose membranes using the iBlot Gel Transfer System (Life Technologies). The Ponceau S (Sigma-Aldrich) was used to check the standard running and transfer conditions. The blots were incubated overnight on a shaker with AQP5 (35 kDa) and β-actin (43 kDa) polyclonal antibodies (Santa Cruz Biotechnology, Inc., Dallas, TX, USA, diluted 1:200, host: rabbit, specificity: mouse, rat and human) in blocking buffer. Antibody binding was detected with the Western Breeze^®^ Chromogenic immunodetection kit (ThermoFisher Scientific. The AQP5 antibody reacted to several bands, including a band at the size of AQP5 (43 kDa). The bands resulting from non-specific bindings of the polyclonal AQP5-antibody were not changed by hormonal or drug treatments.

Images were captured with the EDAS290 imaging system (Csertex Ltd., Budapest, Hungary), and the optical density of each immunoreactive band was determined with Kodak 1D Images analysis software. The β-actin was used for protein normalization for this semi-quantitative methods. Optical densities were calculated as arbitrary units after local area background subtraction.

### 4.5. Statistical Analysis

All experiments were carried out on six animals, and the molecular biology studies were repeated three times. Statistical analyses were performed using Prism 5.0 software (Graph Pad Software, Inc., San Diego, CA, USA). ANOVA Dunnett test or two-tailed unpaired *t* test were used. *p* < 0.05 was considered as a level of significance.

## Figures and Tables

**Table 1:** Parameters of the applied primers and PCR reactions. The real-time reverse transcription polymerase chain reactions were used to determine the changes in mRNA expression. In our studies, the parameters of inventoried TaqMan assays were defined by Life Technologies (ThermoFisher Scientific, Budapest, Hungary).

TaqMan Assays	Assay ID (ThermoFisher Scientific)	Accession Number	Assay Location	Amplicon Length	Annealing Temp. (°C)	Reaction Volume (µL)
AQP5	Rn00562837_m1	NM_012779.1	473	69	60	20
β-Actin	Rn00667869_m1	NM_031144.3	881	91	60	20

## Abbreviations

AQP	Aquaporin
